# Comparison of Intravitreal Dexamethasone Implant and Ranibizumab in Vitrectomized Eyes with Diabetic Macular Edema

**DOI:** 10.1155/2021/8882539

**Published:** 2021-09-10

**Authors:** Jia-Kang Wang, Tzu-Lun Huang, Pei-Yao Chang, Wei-Ting Ho, Yung-Ray Hsu, Fang-Ting Chen, Yun-Ju Chen

**Affiliations:** ^1^Department of Ophthalmology, Far Eastern Memorial Hospital, New Taipei City, Taiwan; ^2^Department of Electrical Engineering, Yuan Ze University, Taoyuan City, Taiwan; ^3^Department of Medicine, National Yang-Ming University, Taipei City, Taiwan; ^4^Department of Medicine, National Taiwan University, Taipei City, Taiwan; ^5^Department of Healthcare Administration and Department of Nursing, Oriental Institute of Technology, New Taipei City, Taiwan

## Abstract

**Purpose:**

This retrospective study aimed to compare the efficacy of intravitreal ranibizumab (IVR) and intravitreal dexamethasone implant (IDI) for pseudophakic vitrectomized eyes with diabetic macular edema (DME) in a single institution.

**Methods:**

Pseudophakic vitrectomized eyes with treatment-naïve center-involved DME were enrolled, with one eye in each patient. They were divided into two groups: one group receiving IDI every 3 to 4 months and another group receiving IVR using 3 monthly plus treat-and-extend injections, all with monthly follow-up for 6 months. Switch of intravitreal drugs or deferred macular laser was not allowed. Primary outcome measures included change in central foveal thickness (CFT) in 1 mm by spectral-domain optical coherence tomography and best-corrected visual acuity (BCVA) at Month 6.

**Results:**

Twenty-two eyes were included in the IDI group and 26 eyes in the IVR group. The baseline demographics, glycosylated hemoglobin level, intraocular pressure (IOP), BCVA, and CFT did not significantly differ (*p* > 0.05). Compared to baseline data, CFT decreased and BCVA improved significantly after either IDI or IVR at Month 6 (*p* < 0.05). Significantly better mean final BCVA (0.38 logMAR vs. 0.62 logMAR, *p*=0.04), more mean visual gain (−0.30 logMAR vs. −0.15 logMAR, *p*=0.02), lower mean final CFT (310.9 *μ*m vs. 384.2 *μ*m, *p*=0.04), and larger mean CFT decrease (−150.0 *μ*m vs. −60.1 *μ*m, *p*=0.03) were found in the IDI group compared to those in the IVR group. A smaller mean treatment number (2.6 vs. 5.6, *p* < 0.001) and higher rate of postinjection ocular hypertension requiring topical hypotensive agent therapy (27.3% vs. 0%, *p*=0.0002) were demonstrated in the IDI group than those in the IVR group.

**Conclusion:**

We concluded that IDI and IVR can both effectively treat vitrectomized eyes with DME. Dexamethasone implants had significantly better visual/anatomical improvement, smaller treatment number, and higher rate of elevated IOP after injection than IVR in pseudophakic vitrectomized eyes with DME in a 6-month period.

## 1. Introduction

Macular edema is an important cause of visual impairment in patients with diabetes. Diabetic macular edema (DME) is associated with ischemia caused by disturbance of microvascular circulation in the diabetic retina [1]. The evidence is that the intraocular vascular endothelial growth factor (VEGF) level is not only elevated in eyes with DME but also proportional to the severity of DME, such as increased macular thickness, enlarged macular volume, and presence of submacular fluid [2]. Ranibizumab (Lucentis®, Novartis Pharma AG, Basel, Switzerland, and Genentech Inc., South San Francisco, CA, USA) is the monoclonal antibody fragment of VEGF-A, which can lower intraocular VEGF level after intravitreal administration in patients with DME [3]. Intravitreal ranibizumab (IVR) can effectively treat DME in randomized controlled or real-life studies [4–9].

Inflammation also plays a crucial role in the formation of DME [1]. Various increased inflammatory cytokines can be detected in the aqueous or vitreous of the eyes with DME, such as monocyte chemoattractant protein 1 (MCP-1), intercellular adhesion molecule 1, interleukin- (IL-) 6, and IL-8. Higher levels of these cytokines are related to more severe macular edema in diabetic eyes [10, 11]. Dexamethasone implants (Ozurdex®, Allergan Inc., Irvine, CA, USA) can slowly release corticosteroids, which reduces both intraocular inflammatory cytokine and VEGF levels after intravitreal injections [12]. Randomized trials or real-world studies proved that intravitreal dexamethasone implant (IDI) decreased DME severity [13–16].

Vitrectomy is required for the removal of vitreous hemorrhage or retinal traction tissue in some patients with proliferative diabetic retinopathy. Postvitrectomy macular edema may develop in these patients with diabetes.

A poor outcome of VEGF inhibitors was often observed following treatment of DME in vitrectomized patients, potentially in response to an enhanced wash-out in eyes without the vitreous barrier or alteration in intravitreal cytokines following vitrectomy [17–21]. Conversely, DME responded well to dexamethasone implants even in these vitrectomized eyes [13]. This retrospective study aimed to compare the clinical behavior between IDI and ranibizumab in vitrectomized patients with DME. To the best of our knowledge, this is the first study involving such subjects.

## 2. Methods

The protocol of the study, which followed the Declaration of Helsinki, was approved by the Institutional Review Board of Far Eastern Memorial Hospital in Taiwan. The study was registered as NCT04089605 at ClinicalTrials.gov on September 13, 2019. We retrospectively enrolled one pseudophakic vitrectomized eye in each patient with treatment-naïve center-involved DME from June 2017 to November 2018 by four surgeons (Wang JK, Chen FT, Hsu YR, and Chen YJ) and with follow-up for 6 months. All patients aged >18 years and had glycosylated hemoglobin (HbA1c) level <10.0%. They presented with best-corrected visual acuity (BCVA) between 20/400 and 20/40, central foveal thickness (CFT) > 300 *μ*m in the 1 mm central macular subfield on spectral-domain optical coherence tomography (SD-OCT, CIRRUS™ HD-OCT 5000, Carl Zeiss Meditec Inc., Dublin, CA, USA), using six radial line scans through the fovea, and macular leakage on fundus fluorescein angiography (HRA2, Heidelberg Engineering GmbH, Germany). The DME pattern can include submacular fluid, cystoid changes, and diffuse macular thickening but exclude accompanying macular traction by the epiretinal membrane or posterior hyaloid. These patients all had proliferative diabetic retinopathy treated by panretinal photocoagulation without silicone oil or gas inside the vitreous cavity, and intraocular surgery was performed at least 3 months ago. We excluded pregnant or nursing women and patients with a history of thromboembolic events or major surgery within the previous 3 months, presence of anterior chamber intraocular lens or subluxated/dislocated posterior chamber intraocular lens, uncontrolled hypertension, known coagulation abnormalities or current use of anticoagulative medication other than aspirin, previous macular photocoagulation or photodynamic therapy, presence of active infectious disease or intraocular inflammation, intraocular pressure (IOP) > 20 mmHg or glaucoma history, or presence of iris neovascularization/vitreous hemorrhage.

Under recommendation by the Ministry of Health and Welfare in Taiwan, we used “patient-doctor shared decision-making” for the selection of DME treatments: IDI or ranibizumab. The process included doctors providing guidelines or advice for DME treatment and the patients choosing the treatment. We provided advice for patients' selection of management for DME according to the 2017 EURETINA guidelines and outcomes of one previous ranibizumab and dexamethasone implants head-to-head multicenter randomized comparison study (MAGGIORE study) [22, 23]. The doctor's suggestions were presented as follows: (1) comparably and significantly visual and anatomical improvement can be achieved following intravitreal injections between ranibizumab and dexamethasone implants in nonvitrectomized eyes, and both treatments were more effective than macular laser. (2) Significantly less injections were required in dexamethasone implant than those in ranibizumab. (3) An increase in IOP that was needed for topical medical control occurred in 10%–30% of patients after IDI but in fewer patients after ranibizumab treatment [13–16, 23]. (4) Low incidence of thromboembolic events may be noted after IVR injection in patients with risks of cerebrovascular accidents or cardiovascular diseases but not after dexamethasone implant injections.

Following patients' selection of intravitreal agents, we would submit the reimbursement of National Health Insurance in Taiwan. The reimbursement provided a maximum of eight injections of ranibizumab 0.5 mg in 0.05 mL or five injections of dexamethasone implants 0.7 mg within 5 years, and no drug switch was allowed. Following reimbursement approval, the patients provided written informed consent for intravitreal injections. As for IVR injection, we used OCT-guided treat-and-extend protocol for DME treatment after modifying the settings of the TREX-DME study [4]. The regimen included three monthly loading doses; then, the treatment injection interval was extended one month if CFT was <300 *μ*m without obvious submacular fluid and intramacular cysts. The injection interval shortened one month if CFT was >300 *μ*m or obvious fluid and/or cysts were present. The patients were intentionally injected at most every 3 months, even in the absence of DME. The eyes underwent dexamethasone intravitreal implant injections at baseline and every 3 or 4 months thereafter. Dexamethasone implants were reinjected in a minimum of 3-month intervals if macular edema persisted or recurred with CFT >300 *μ*m or presence of apparent submacular fluid and/or intramacular cysts. If DME subsided with CFT <300 *μ*m without accompanying fluid and cysts, a repeated injection was mandatory in a maximum of 4-month interval. Deferred macular laser was not added in any patient in the two groups. The examinations of slit lamp, BCVA in Snellen chart (converted into logMAR and EDTRS letters for statistical comparison), IOP via pneumotonometer (CT-80, Topcon Inc., Tokyo, Japan), SD-OCT of the macula, and dilated fundus were performed every month up to 6 months of follow-up. The follow-up SD-OCT scans used the baseline scan as a reference. If IOP was >20 mmHg after injection during the follow-up visits, topical hypotensive agents were provided. Visual testing was performed in the same room at each visit. Primary outcome measures included changes in CFT and BCVA at Month 6. Injection number, BCVA, CFT, postinjection complications, and IOP were recorded and compared with Wilcoxon signed-rank test within the group and Wilcoxon rank-sum test between groups. Fisher's exact test was used for categorical comparison between groups. A *p* value <0.05 was considered significant.

## 3. Results

A total of 48 pseudophakic vitrectomized eyes of 48 patients with diabetes with center-involved macular edema were included. 32 phakic eyes initially received vitrectomy and subsequent uneventful cataract surgery, and another 16 pseudophakic eyes underwent vitrectomy. Vitrectomy was performed for all these eyes for severe proliferative diabetic retinopathy complicated with vitreous hemorrhage in 28 eyes, tractional retinal detachment in seven eyes, active epiretinal fibrovascular proliferation in five eyes, taut posterior hyaloid macular traction in two eyes, and macular pucker in six eyes. The macular internal limiting membrane was not peeled during vitrectomy. All eyes had persistent diabetic macular edema >3 months after the last surgery.

Of these 48 patients, 26 eyes of 26 patients received IVR, and 22 eyes of 22 patients received dexamethasone implant. Baseline clinical data and all comparable data between the two groups, including age, sex, HbA1c level, and mean baseline BCVA/CFT/IOP, are presented in Table 1 (*p* > 0.05).

In the dexamethasone implant group, the mean BCVA significantly improved from Month 2 (0.47 ± 0.22 logMAR) to Month 6 (0.38 ± 0.38 logMAR) after dexamethasone intravitreal implant treatment (*p* < 0.05), except for Month 1 (0.58 ± 0.29 logMAR) (*p*=0.24), compared to baseline. One-line loss of BCVA was noted in 13.6% of patients because of decreased but persistent intraretinal cyst and/or submacular fluid on SD-OCT after dexamethasone implant injection. The mean CFT significantly decreased from 325.1 ± 52.2 *μ*m at Month 1 to 310.9 ± 128.8 *μ*m at Month 6 following dexamethasone implant compared to the mean baseline CFT (*p* < 0.05). The mean changes from baseline to final BCVA, percentage of patients with final BCVA ≥20/40, percentage of BCVA gain ≥3 lines, and mean decrease from baseline to final CFT in the dexamethasone implant group are shown in Table 2.

In the ranibizumab group, the mean BCVA significantly improved from Month 2 (0.67 ± 0.35 logMAR) to Month 6 (0.62 ± 0.41 logMAR) after ranibizumab treatment (*p* < 0.05), except for Month 1 (0.71 ± 0.45 logMAR) (*p*=0.29), compared to baseline. One-line loss of BCVA was noted in 23.1% of patients, owing to reduced but some residual intraretinal cysts and/or submacular fluid on SD-OCT after dexamethasone implant injection. The mean CFT significantly decreased from 385.2 ± 115.9 *μ*m at Month 1 to 384.2 ± 108.6 *μ*m at Month 6 following IDI compared to the mean baseline CFT (*p* < 0.05). The mean changes from baseline to final BCVA, percentage of patients with final BCVA ≥20/40, percentage of BCVA gain ≥3 lines, and mean decrease from baseline to final CFT in the ranibizumab group are shown in Table 2.

**Figure 1:**
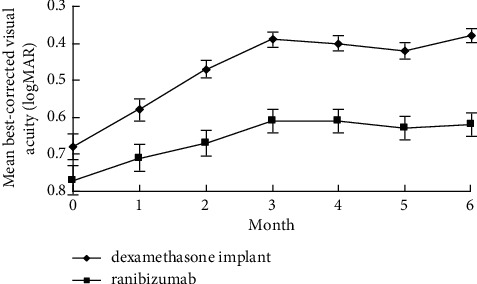
Changes of best-corrected visual acuity from baseline to Month 6 in pseudophakic vitrectomized eyes with diabetic macular edema treated by intravitreal dexamethasone implant or ranibizumab.

**Figure 2:**
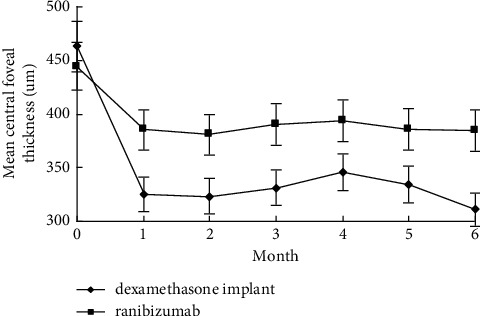
Changes of central foveal thickness from baseline to Month 6 in pseudophakic vitrectomized eyes with diabetic macular edema treated by intravitreal dexamethasone implant or ranibizumab.

**Figure 3:**
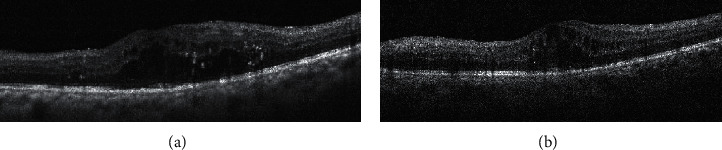
Diabetic macular edema persisting after three monthly intravitreal ranibizumab in a vitrectomized eye: (a) macular optical coherence tomography (OCT) before injections; (b) macular OCT three months after injections.

**Figure 4:**
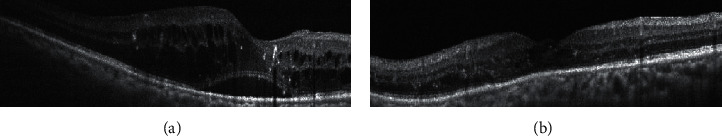
Diabetic macular edema disappearing after single intravitreal dexamethasone implant in a vitrectomized eye: (a) macular optical coherence tomography (OCT) before injections; (b) macular OCT three months after injections.

Final BCVA, BCVA gain, percentage of final BCVA ≥20/40, and percentage of BCVA gain ≥3 lines were all significantly better in the dexamethasone implant group than those in the ranibizumab group (*p* < 0.05). Thinner final CFT and significantly decreased CFT were observed in eyes receiving dexamethasone implants compared to eyes receiving IVR (*p* < 0.05). The percentage of BCVA loss ≥1 line was lower in the dexamethasone implant group than that in the ranibizumab group (*p*=0.02). At all time points from Month 1 to Month 6, mean BCVA and CFT were significantly superior in the dexamethasone implant group (*p* < 0.05), except for mean BCVA at Month 1, which was comparable between the two groups (*p*=0.27) (Figures 1–4).

The mean dexamethasone implant injection number at 6 months was 2.6 ± 0.5, which was significantly lower than 5.6 ± 0.9 in the ranibizumab group (*p* < 0.001) (Table 2). Ten eyes required only two injections of dexamethasone implant, and 12 eyes required three injections during the 6-month study period. In the ranibizumab group, ranibizumab was injected 4 times in 3 eyes, 5 times in 10 eyes, 6 times in 8 eyes, and 7 times in 5 eyes from Months 0 to 6.

The injections were well tolerated in all patients. No serious ocular or systemic complications were observed in all eyes, such as thromboembolic events, retinal detachment, infectious endophthalmitis, anterior chamber migration of the dexamethasone implant, and intractable IOP elevation requiring glaucoma incisional surgery. Only temporary elevation of IOP was found in 6 of 22 eyes (27.3%) after dexamethasone implant administration, but not after ranibizumab injection (*p*=0.0002). These six eyes developed maximal IOP >20 mmHg after dexamethasone implant treatment and required medical control, with a mean IOP of 26.3 ± 4.7 mmHg between 1 and 3 months after injection. Maximal IOP elevation of 4 eyes ranging from 21 to 30 mmHg was well controlled by topical brimonidine and that of 2 eyes ranging from 31 to 34 mmHg was controlled by a fixed combination of topical brimonidine and timolol. No patient needed surgical treatment of postinjection increased IOP. The final IOP of 19.7 ± 4.2 mmHg in the dexamethasone implant group was similar to that of the ranibizumab group (18.9 ± 3.2 mmHg) (*p*=0.37). Other common side effects were local hyperemia or subconjunctival hemorrhage at the injection site.

## 4. Discussion

In the study, there were two groups of pseudophakic vitrectomized patients with DME receiving IDI or ranibizumab independently. Both regimens were effective in macular thickness reduction and visual increase after 6-month treatment. Based on matched baseline factors, the dexamethasone implant injections resulted in significantly better mean final BCVA (0.38 logMAR vs. 0.62 logMAR, *p*=0.04), more mean visual gains (−0.30 logMAR vs. −0.15 logMAR, *p*=0.02), lower mean final CFT (310.9 *μ*m vs. 384.2 *μ*m, *p*=0.04), larger mean CFT decrease (−150.0 *μ*m vs. −60.1 *μ*m, *p*=0.03), smaller mean treatment number (2.6 vs. 5.6, *p* < 0.001), and higher rate of postinjection ocular hypertension requiring topical hypotensive agent therapy (27.3% vs. 0%, *p*=0.0002) than ranibizumab injection.

Vitrectomy in patients with diabetes replaces the vitreous gel with liquid, which can change the pharmacokinetics of intravitreal drugs, such as ranibizumab in the form of solution in several ways. First, the drug solution diluted by the vitreous fluid after intravitreal injection probably results in poorer efficacy. Second, the drug liquid is not trapped in the vitreous gel but evenly distributes the vitreous fluid. According to the Stokes–Einstein law, molecular diffusion is faster in saline solution than in vitreous humor [24]. The fact caused higher clearance and shorter action duration of the medication in the vitrectomized eyes than those in the nonvitrectomized eyes. The rabbit vitrectomized eyes showed half-life of ranibizumab shortened compared with nonvitrectomized eyes after IVR in previous studies [19, 20]. The half-life of ranibizumab in the aqueous humor was 2.3 days in the nonvitrectomized group, which was longer than 1.4 days in the vitrectomized group after IVR in macaque eyes in another previous animal study [25]. Moreover, the vitreous body is removed after vitrectomy, which may change the different intraocular cytokine levels. An animal study showed decreased vitreous VEGF level, owing to the shortened half-life of vitreous VEGF in vitrectomized eyes compared to that in nonvitrectomized eyes [26]. Two previous studies revealed that vitrectomy decreased aqueous VEGF level and increased vitreous MCP-1 and IL-6 levels [17, 18]. The fact implied that DME after vitrectomy was more associated with inflammatory cytokines and less with VEGF. All these three factors can lead to poor performance of IVR for postvitrectomy macular edema in patients with diabetes.

Laugesen et al. found that ranibizumab injections could only reduce macular thickness but could not improve visual acuity in patients with DME who underwent vitrectomy [27]. Koyanagi et al. compared the difference in the efficacy of ranibizumab for DME between vitrectomized and nonvitrectomized eyes [28]. Visual acuity did not significantly increase, but the macula became clearly less edematous after ranibizumab injection in the vitrectomized group. They demonstrated worse final visual acuity and higher macular thickness after IVR in vitrectomized eyes than those in nonvitrectomized eyes. Our previous study also enrolled patients with diabetes with or without preceding vitrectomy treated by ranibizumab treatment for 6 months [29]. Although IVR showed efficacy for DME in both vitrectomized and nonvitrectomized patients, ranibizumab had a significantly better ability to improve DME in nonvitrectomized eyes than that in vitrectomized eyes. In the post hoc analysis of protocol I in DRCR.net research, IVR demonstrated comparable visual outcomes but suboptimal anatomical responses for DME in vitrectomized eyes compared to those in nonvitrectomized eyes within the first year [30]. Significant visual improvement in our study was analogous to the outcomes of two previous studies using IVR for DME after vitrectomy [29, 30].

Dexamethasone implants are composed of dexamethasone wrapped with specially designed NOVADUR®. The biodegradable NOVADUR® can slowly deliver dexamethasone and dissolve within 6 months and is theoretically not affected by the surrounding microenvironment, such as vitreous gel and fluid. The hypothesis was proven in a previous animal experiment [21]. The authors discovered dexamethasone implants existed as long as 31 days in both vitrectomized and nonvitrectomized groups of rabbit eyes. Both groups also shared the same pharmacokinetics after intravitreal placement of dexamethasone implants. Comparable visual/anatomical outcomes and rates of postinjection ocular hypertension were noted between vitrectomized and nonvitrectomized eyes after dexamethasone implant for DME in five previous clinical studies [31–37]. Dexamethasone implant injections also resulted in noticeable visual gain and foveal thickness reduction in vitrectomized patients with DME. As for elevated IOP after IDI, 27.3% of patients required medical control, similar to a rate of 17%–28.8% in the vitrectomized eyes with DME reported in previous studies [31, 33, 35]. Incisional glaucoma surgery was not needed for the treatment of IOP increase after dexamethasone implant as previously reported studies [32–36].

A meta-analysis based on four randomized clinical trials demonstrated that intravitreal anti-VEGF agents and dexamethasone implants shared similar efficacy for DME control [37]. Dexamethasone implants reported similar final visual acuity to anti-VEGF agents to treat DME in a summary of real-life observational studies [38]. A multicenter randomized trial (MAGGIORE study) compared the clinical performance between IDI and ranibizumab for nonvitrectomized eyes with DME during the 1-year period [23]. The mean average change of BCVA from baseline through Month 12 was 4.3 and 7.6 letters in the dexamethasone implant and ranibizumab groups, respectively, in all patients, whereas 4.6 and 6.6 letters individually in pseudophakic eyes. The authors defined the noninferiority of dexamethasone implant to ranibizumab in visual gain during the 1-year follow-up. In this study, different from the visual results of the MAGGIORE study in nonvitrectomized patients, the mean visual gains were 17.2 letters in the dexamethasone implant group, superior to 6.6 letters in the ranibizumab group despite the significant visual improvement observed in both groups in these pseudophakic vitrectomized eyes of our study [23]. The relatively poorer performance of ranibizumab in vitrectomized eyes can be explained as the results of vitreous saline dilution of ranibizumab, aggravated ranibizumab turn-over rate, and decreased intraocular VEGF level after vitrectomy [17, 19, 20]. Dexamethasone implants gradually released corticosteroids inside the vitreous liquid and suppressed inflammatory cytokines occurring after vitrectomy, which possibly led to greater efficacy of dexamethasone implants compared to ranibizumab in vitrectomized eyes with DME [18, 21]. The number of dexamethasone implant injections was significantly fewer than that of ranibizumab in this study because the implants had a longer duration of action even in the vitrectomized patients. Medication to lower IOP was required in nearly 1/4 of patients receiving dexamethasone implant injections, but it was not needed in any of the eyes receiving IVR.

To the best of our knowledge, no publication compared the clinical outcome of dexamethasone implants and ranibizumab for pseudophakic vitrectomized patients with DME. The study limitations were the relatively small sample size and single-center design in a short-term period. Some bias may occur in the study setting. A large-scale, multicenter, and long-term trial will be required to justify or correlate the results of our study.

In conclusion, fewer intravitreal injections of dexamethasone implants can achieve better visual and anatomical improvement for macular edema in diabetic pseudophakic patients after vitrectomy compared to IVR administration. However, dexamethasone implant injection can cause more controllable IOP elevation than IVR treatment.

## Figures and Tables

**Table 1:** Comparison of baseline data of intravitreal dexamethasone implant and ranibizumab in pseudophakic vitrectomized eyes for diabetic macular edema.

	Dexamethasone implant (*n* = 22)	Ranibizumab (*n* = 26)	*p* value
Age (years)	59.9 ± 10.4	59.9 ± 9.4	0.99
Gender (male: female)	16 : 6	17 : 9	0.09
HbA1c (%)	8.1 ± 1.9	7.9 ± 2.1	0.25
Central foveal thickness (*μ*m)	462.9 ± 136.7	444.4 ± 118.5	0.63
Best-corrected visual acuity (logMAR)	0.68 ± 0.35	0.77 ± 0.41	0.41
Intraocular pressure (mmHg)	16.2 ± 4.8	17.9 ± 5.1	0.42

HbA1c: glycosylated hemoglobin.

**Table 2:** Comparison of clinical data after 6-month treatment of intravitreal dexamethasone implant and ranibizumab in pseudophakic vitrectomized eyes for diabetic macular edema.

	Dexamethasone implant (*n* = 22)	Ranibizumab (*n* = 26)	*p* value
Final BCVA (logMAR)	0.38 ± 0.38	0.62 ± 0.41	0.04^*∗*^
Changes in BCVA (logMAR)	−0.30 ± 0.36	−0.15 ± 0.41	0.02^*∗*^
Changes in BCVA (ETDRS letters)	17.23 ± 15.32	6.62 ± 14.02	0.03^*∗*^
Final BCVA ≥20/40	14/22 (63.6%)	6/26 (23.1%)	0.008^*∗*^
BCVA gains ≥3 lines	13/22 (59.1%)	7/26 (26.9%)	0.03^*∗*^
BCVA loss ≥1 line	3/22 (13.6%)	6/26 (23.1%)	0.02^*∗*^
Final CFT (*μ*m)	310.9 ± 128.8	384.2 ± 108.6	0.04^*∗*^
Changes in CFT (*μ*m)	−150.0 ± 131.1	−60.1 ± 110.2	0.03^*∗*^
Injection number	2.6 ± 0.5	5.6 ± 0.9	<0.001^*∗*^
Final intraocular pressure (mmHg)	19.7 ± 4.2	18.9 ± 3.2	0.37

BCVA: best-corrected visual acuity; CFT: central foveal thickness. ^*∗*^*P* < 0.05.

## Data Availability

All data generated or analyzed during this study are included in this published article in the supplementary files.

## Abbreviations

IVR: Intravitreal ranibizumab
IDI: Intravitreal dexamethasone implant
DME: Diabetic macular edema
CFT: Central foveal thickness
BCVA: Best-corrected visual acuity
IOP: Intraocular pressure
VEGF: Vascular endothelial growth factor
MCP-1: Monocyte chemoattractant protein 1
ICAM-1: Intercellular adhesion molecule 1
IL-6: Interleukin-6
IL-8: Interleukin-8
SD-OCT: Spectral-domain optical coherence tomography.

## Supplementary Materials

Supplementary Materials The supplementary data was the raw data of the 2 separate groups with diabetic macular edema in vitrectomized eyes undergoing intravitreal ranibizumab and dexamethasone implants, respectively.
