# Supplementary material for: Comparison of Intravitreal Dexamethasone Implant and Ranibizumab in Vitrectomized Eyes with Diabetic Macular Edema
Source: J Ophthalmol. 2021 Sep 10;2021:8882539. doi: 10.1155/2021/8882539 (PMC8448602; doi:10.1155/2021/8882539)
Supplement: Supplementary Materials — The supplementary data was the raw data of the 2 separate groups with diabetic macular edema in vitrectomized eyes undergoing intravitreal ranibizumab and dexamethasone implants, respectively. [file 8882539.f1.pdf]

No.	age	sex	M0 VA	logMAR	letter	M0 CFT	M1 VA	logMAR
1	59	M	0.1	1	35	393	0.16	0.8
2	68	M	0.2	0.7	50	407	0.2	0.7
3	65	M	0.4	0.4	65	371	0.5	0.3
4	49	F	0.3	0.52	59	405	0.7	0.15
5	67	F	0.16	0.8	45	426	0.5	0.3
6	65	M	0.1	1	35	713	0.2	0.7
7	39	M	0.4	0.4	65	393	0.4	0.4
8	69	M	0.1	1	35	757	0.3	0.52
9	54	F	0.2	0.7	50	399	0.2	0.7
10	72	M	0.2	0.7	50	353	0.3	0.52
11	61	M	0.025	1.6	5	650	0.1	1
12	44	M	0.05	1.3	20	712	0.2	0.7
13	67	F	0.4	0.4	65	310	0.5	0.3
14	51	M	0.16	0.8	45	696	0.2	0.7
15	48	M	0.2	0.7	50	414	0.4	0.4
16	49	m	0.7	0.15	77	375	0.9	0.05
17	69	m	0.2	0.7	50	461	0.3	0.52
18	49	m	0.2	0.7	50	421	0.2	0.7
19	76	F	0.05	0.13	20	408	0.05	1.3
20	76	F	0.4	0.4	65	349	0.2	0.7
21	64	M	0.6	0.22	73	334	0.4	0.4
22	56	M	0.2	0.7	50	438	0.1	1
mean	59.8636	16M		0.68273	48.1364	462.955		0.58455
median	62.5							
SD	10.4111	6F		0.35056	17.4676	136.704		0.28833
vs M0								0.23894

VT OZD 22 eyes

1	77	m	0.4	0.4	65	323	0.4	0.4
2	52	m	0.3	0.52	59	312	0.5	0.3
3	61	m	0.3	0.52	59	498	0.4	0.4
4	69	m	0.3	0.52	59	417	0.3	0.52
5	67	m	0.16	0.8	45	596	0.16	0.8
6	52	f	0.4	0.4	65	470	0.4	0.4
7	64	m	0.3	0.52	59	484	0.5	0.3
8	61	m	0.3	0.52	59	481	0.2	0.7
9	61	m	0.16	0.8	45	314	0.16	0.8
10	53	f	0.16	0.8	45	501	0.3	0.52
11	66	m	0.3	0.52	59	431	0.3	0.52
12	75	m	0.05	1.3	20	443	0.05	1.3
13	67	m	0.1	1	35	458	0.2	0.7

14	58	m	0.4	0.4	65	421		0.5	0.3
15	60	m	0.3	0.52	59	277		0.4	0.4
16	54	f	0.025	1.6	5	413		0.1	1
17	66	m	0.3	0.52	59	384		0.3	0.52
18	60	f	0.3	0.52	59	734		0.16	0.8
19	75	f	0.025	1.6	5	467		0.1	1
20	39	m	0.5	0.3	70	380		0.5	0.3
21	56	m	0.05	1.3	20	365	CF/15c		2.3
22	52	f	0.3	0.52	59	319		0.3	0.52
23	39	m	0.4	0.4	65	517		0.2	0.7
24	57	f	0.025	1.6	5	787		0.025	1.6
25	52	f	0.05	1.3	20	443		0.1	1
26	64	f	0.1	1	35	320		0.5	0.3
mean	59.8846	17M		0.77692	46.1538	444.423			0.70769
SD	9.35754	9F		0.41185	20.5926	118.467			0.45364
vs M0									0.29127
VT Lucentis 26 eyes									
OZD vs Lucentis	0.99435			0.4064	0.72549	0.62919			0.27089

M1 CFT	M2 VA	logMAR	M2 CFT	M3 VA	logMAR	M3 CFT	M4 VA	logMAR	M4 CFT
345	0.16	0.8	336	0.2	0.7	299	0.2	0.7	313
401	0.3	0.52	383	0.3	0.52	399	0.2	0.7	430
346	0.6	0.22	299	0.6	0.22	366	0.5	0.3	372
369	0.7	0.15	400	0.6	0.22	444	0.7	0.15	350
246	0.6	0.22	223	0.6	0.22	291	0.6	0.22	280
355	0.6	0.22	343	0.5	0.3	287	0.2	0.7	355
372	0.5	0.3	358	0.9	0.05	374	0.4	0.4	372
213	0.4	0.4	199	0.3	0.52	291	0.3	0.52	383
248	0.2	0.7	250	0.2	0.7	252	0.2	0.7	250
338	0.2	0.7	329	0.2	0.7	343	0.2	0.7	353
411	0.2	0.7	352	0.4	0.4	313	0.7	0.15	411
268	0.4	0.4	250	0.3	0.52	280	0.3	0.52	290
299	0.6	0.22	280	0.8	0.1	378	0.4	0.4	330
289	0.3	0.52	250	0.3	0.52	311	0.5	0.3	463
335	0.4	0.4	328	0.5	0.3	340	0.6	0.22	330
321	0.1	1	317	0.5	0.3	367	0.5	0.3	350
323	0.2	0.7	258	0.6	0.22	259	0.9	0.05	352
362	0.3	0.52	422	0.6	0.22	421	0.7	0.15	372
294	0.4	0.4	370	0.16	0.8	391	0.6	0.22	313
316	0.4	0.4	397	0.4	0.4	324	0.5	0.3	288
292	0.3	0.52	399	0.4	0.4	297	0.4	0.4	340
410	0.4	0.4	372	0.7	0.15	255	0.6	0.22	299
325.136		0.47318	323.409		0.38545	331		0.37818	345.2727
52.2262		0.21552	62.4985		0.2078	53.8466		0.20758	49.34128
0.00058		0.02385	0.00179		0.00162	0.00147		0.00122	0.000395

341	0.3	0.52	307	0.3	0.52	362	0.4	0.4	335
399	0.6	0.22	286	0.7	0.15	278	0.8	0.1	222
298	0.5	0.3	247	0.6	0.22	247	0.7	0.15	240
375	0.3	0.52	380	0.5	0.3	381	0.5	0.3	436
434	0.2	0.7	377	0.05	1.3	411	0.16	0.8	366
495	0.4	0.4	454	0.6	0.22	482	0.6	0.22	528
469	0.5	0.3	452	0.7	0.15	463	0.7	0.15	460
424	0.2	0.7	405	0.3	0.52	386	0.3	0.52	370
280	0.1	1	381	0.16	0.8	290	0.16	0.8	399
404	0.3	0.52	398	0.2	0.7	345	0.3	0.52	336
293	0.4	0.4	295	0.5	0.3	377	0.7	0.15	325
333	0.05	1.3	441	0.05	1.3	372	0.1	1	351
328	0.16	0.8	347	0.2	0.7	325	0.2	0.7	370

390	0.6	0.22	358	0.7	0.15	377	0.8	0.1	344
240	0.5	0.3	238	0.5	0.3	232	0.5	0.3	331
400	0.05	1.3	399	0.1	1	403	0.2	0.7	499
324	0.2	0.7	447	0.4	0.4	312	0.2	0.7	598
634	0.16	0.8	718	0.2	0.7	617	0.1	1	666
443	0.1	1	521	0.16	0.8	597	0.1	1	514
285	0.6	0.22	385	0.9	0.05	368	0.5	0.3	367
328	0.2	0.7	500	0.5	0.3	626	0.6	0.22	367
314	0.4	0.4	315	0.5	0.3	305	0.7	0.15	378
544	0.2	0.7	535	0.4	0.4	662	0.3	0.52	462
732	0.05	1.3	205	0.05	1.3	390	0.05	1.3	415
284	0.05	1.3	295 ND		2.3	305 ND		2.3	343
223	0.2	0.7	217	0.5	0,3	228	0.6	1.3	221
385.154		0.66615	380.885		0.6072	390.038		0.60385	393.9615
115.936		0.348	111.322		0.50479	117.91		0.49566	103.6998
0.00014		0.02441	0.02535		0.0479	0.03764		0.04151	0.045567
0.02594		0.02659	0.03347		0.05736	0.03125		0.0459	0.04415

M5 VA	logMAR	M5 CFT	M6 VA	logMAR	letter	M6 CFT	inj number	
0.2	0.7	351	0.05	1.3	20	382	3	
0.2	0.7	470	0.16	0.8	45	384	3	
0.4	0.4	389	0.3	0.52	59	400	3	
0.7	0.15	322	0.6	0.22	73	213	3	
0.6	0.22	265	0.7	0.15	77	253	3	
0.6	0.22	343	0.5	0.3	70	298	3	
0.5	0.3	358	0.5	0.3	70	358	3	
0.3	0.52	430	0.2	0.7	50	510	3	
0.2	0.7	250	0.2	0.7	50	347	3	
0.2	0.7	322	0.2	0.7	50	252	3	
0.2	0.7	352	0.05	1.3	20	748	3	
0.2	0.7	544	0.8	0.1	65	250	3	
0.5	0.3	299	0.8	0.1	80	280	3	
0.3	0.52	289	0.4	0.4	65	250	2	
0.5	0.3	298	0.6	0.22	73	380	2	
0.3	0.52	336	0.8	0.1	80	255	2	
0.6	0.22	300	0.9	0.05	82	223	2	
0.2	0.7	320	1	0	85	344	2	
0.7	0.15	315	0.7	0.15	77	189	2	
0.8	0.1	303	0.8	0.1	80	177	2	
0.7	0.15	289	0.9	0.05	82	158	2	
0.6	0.22	211	1	0	85	190	2	
	0.417727	334.364		0.375455	65.36364	310.9545	2.59090909	3 inj 12
	0.223951	71.5374		0.380547	18.68044	128.8069	0.49166608	2 inj 10
	0.000577	0.00011		0.000592		0.000115		

0.4	0.4	341	0.4	0.4	65	334	4
0.9	0.05	319	0.4	0.4	65	221	4
0.8	0.1	215	0.4	0.4	65	223	4
0.5	0.3	361	0.5	0.3	70	345	5
0.16	0.8	372	0.05	1.3	20	364	5
0.6	0.22	431	0.3	0.52	59	563	5
0.7	0.15	465	0.4	0.4	65	452	5
0.3	0.52	358	0.3	0.52	59	367	5
0.16	0.8	359	0.3	0.52	59	345	5
0.5	0.3	446	0.6	0.22	73	314	5
0.2	0.7	449	0.8	0.1	80	342	5
0.05	1.3	289	0.1	1	35	440	5
0.3	0.52	389	0.3	0.52	59	363	5

0.9	0.05	362	1	0	50	343	6	
0.2	0.7	331	0.2	0.7	50	341	6	
0.05	1.3	415	0.1	1	35	399	6	
0.2	0.7	454	0.2	0.7	50	444	6	
0.05	1.3	643	0.05	1.3	20	621	6	
0.05	1.3	494	0.05	1.3	20	684	6	
0.5	0.3	478	0.4	0.4	65	494	6	
0.5	0.3	344	0.7	0.15	77	388	6	
0.3	0.52	299	0.3	0.52	59	387	7	
0.3	0.52	395	0.3	0.52	59	367	7	
0.05	1.3	528	0.05	1.3	20	270	7	
0.05	1.3	291	0.05	1.3	20	334	7	
0.3	0.52	204	0.6	0.22	73	245	7	
	0.625769	385.846		0.615769	52.76923	384.2308	5.57692308	4 inj 3
	0.423658	117.643		0.402551	19.06327	108.5535	0.92707468	5 inj 10
	0.031064	0.00577		0.048436		0.044047		6 inj 8
	0.039771	0.04081		0.043424	0.028955	0.045435	8.5004E-17	

